# Longitudinal osmotic and neurometabolic changes in young rats with chronic cholestatic liver disease

**DOI:** 10.1038/s41598-020-64416-3

**Published:** 2020-05-05

**Authors:** Veronika Rackayova, Olivier Braissant, Anne-Laure Rougemont, Cristina Cudalbu, Valérie A. McLin

**Affiliations:** 1 Laboratory of Functional and Metabolic Imaging (LIFMET), Ecole Polytechnique Fédérale de Lausanne (EPFL), Lausanne, Switzerland; 2 Service of Clinical Chemistry, University of Lausanne and University Hospital of Lausanne, Lausanne, Switzerland; 3 Division of Clinical Pathology, University Hospitals Geneva, Geneva, Switzerland; 4 Centre d’Imagerie Biomédicale (CIBM), Ecole Polytechnique Fédérale de Lausanne (EPFL), Lausanne, Switzerland; 5 Swiss Pediatric Liver Center, University Hospitals Geneva, and Department of Pediatrics, Obstetrics and Gynecology, Faculty of Medicine, University of Geneva, Geneva, Switzerland

**Keywords:** Portal hypertension, Hepatic encephalopathy

## Abstract

Type C hepatic encephalopathy (type C HE) is increasingly suspected in children with chronic liver disease (CLD), and believed to underlie long-term neurocognitive difficulties. The molecular underpinnings of type C HE in both adults and children are incompletely understood. In the present study we combined the experimental advantages of *in vivo* high field ^1^H magnetic resonance spectroscopy with immunohistochemistry to follow longitudinally over 8 weeks the neurometabolic changes in the hippocampus of animals having undergone bile duct ligation as pups. Rats who develop CLD early in life displayed pronounced neurometabolic changes in the hippocampus characterized by a progressive increase in glutamine concentration which correlated with plasma ammonia levels and a rapid decrease in brain myo-inositol. Other neurometabolic findings included a decrease in other organic osmolytes (taurine, choline-containing compounds and creatine), ascorbate and glutamate. At the cellular level, we observed an increase in glial fibrillary acidic protein (GFAP) and aquaporin 4 (AQP4) expression in the hippocampus at 4 weeks post bile duct ligation (BDL), together with astrocytic morphological alterations. These findings differ from observations in the brain of adult rats following BDL, and are in keeping with the commonly accepted theory of age-dependent vulnerability.

## Introduction

In children with CLD, neurocognitive impairment is described before liver transplantation^1,2^, and these abnormalities do not resolve following liver transplant^3,4^. Further, there is evidence that children with CLD may have unique neurocognitive vulnerabilities compared to children with other chronic conditions or to pediatric recipients of other solid organ transplants^5^.

It is recognized that patients with urea cycle defects who experience acute and severe hyperammonemia in infancy have long lasting neurological impairment^6,7^. This is in contrast to the reversibility of mental status and neurological changes of adults with acute bouts of hyperammonemia in the setting of acute liver failure^8^. At a cellular level, it has been shown in 3D organotypic brain cell cultures that ammonium exposure impairs axonal growth in developing brain cells while more mature neurons remain unaffected^6^, confirming the clinical impression that the developing central nervous system (CNS) appears particularly vulnerable to hyperammonemia^8^. Moreover, there is the additional variable of an immature blood brain barrier (BBB) early in life^9^, likely contributing further to this time-sensitive vulnerability.

Although type C HE is increasingly sought and described in patients with CLD, its molecular underpinnings are still incompletely understood. CLD is known to provoke neurometabolic changes, mainly due to the challenge of detoxifying an increased ammonium load to the brain. Brain glutamine (Gln) and decreased myo-inositol (mIns) and choline containing compounds (total choline - tCho) have been shown both in human subjects^10,11^ and animals with CLD^12–14^. However, there are few reports of ^1^H magnetic resonance spectroscopy (MRS) studies in children with CLD^15–17^ as well as only few adult animal studies^12–14^. To date, ^1^H-MRS studies in patients with CLD have only been performed on MR scanners up to 3 Tesla (T), allowing the measurement of a limited number of metabolites, thereby limiting our understanding of the molecular events in the CNS of patients with CLD. Studying type C HE in an animal model at higher resolution offers more granular insight into the longitudinal, molecular changes occurring during HE progression^18^. High resolution in ^1^H-MRS allows for the identification of more metabolites, which is warranted to tease out the impact of CLD from developmental processes and to detect subtle, early changes.

What happens at the molecular and cellular levels to the brain of young subjects with CLD is understudied, as cognitive and motor delays have historically be ascribed to chronic illness, not focusing on the specific risks of CLD on brain development, something which this study aimed to address longitudinally in an animal model of CLD acquired at a young age. Therefore, the novelty of the present study was to use the experimental advantages of high field ^1^H-MRS to analyse longitudinally the hippocampus of rats having undergone bile duct ligation (BDL) as pups and to compare these findings to those of a similar study from our lab in adult rats, the underlying hypothesis being that the pups would display more significant neurometabolic changes than adults.

## Methods

### Study design

All animal experiments were conducted according to federal and local ethical guidelines, and the protocols were approved by the local Committee on Animal Experimentation for the Canton de Vaud, Switzerland (VD2761).

Twelve (12) male Wistar pups underwent BDL and 7 animals were sham operated on postnatal day 21 (P21). Blood sampling and *in vivo* brain ^1^H-MRS scans were performed at week 2, 4, 6 and 8 post-BDL. Behavioural tests were performed to measure motor activity at week 4, 6 and 8 post-BDL, before MRS scans.

To approximate rat- and human- brain development, we used a previously published neuroinformatics approach^19,20^. According to this study, overall brain development of the P21 rat (day of BDL surgery) is the equivalent of an 8 month old human, while P77 old rats (end of study: week 8 post-BDL or post coital age 98 days) approximate an 8.5 year old human (http://translatingtime.org/translate).

### Validation of CLD and type C HE induced by BDL in P21 rats

#### Biochemical measurements

Plasma samples were analysed using Reflotron® System for glucose, Integra® 400 Plus for ammonium and COBAS® 8000 for total bilirubin as markers of biliary obstruction and liver function (Roche, Switzerland).

#### Behavioural tests

Locomotor activity was assessed in the open field (OF) test^18^, to confirm the presence of motor deficits (characteristic of type C HE)^21,22^.

#### *In vivo*^1^H-MRS

*In vivo* ^1^H-MRS was performed as previously described^18^. Measurements were conducted on a horizontal 9.4 T MR-system using SPECIAL sequence (TE = 2.8 ms) in a volume of interest (2 × 2.8 × 2 mm^3^) placed in dorsal hippocampus. The rationale for studying the hippocampus was two fold: 1) it is implicated in learning and memory, both of which are impaired in humans with type C HE^23^ and 2) it is technically easily accessible and offers an excellent signal at 9.4 T. 18 brain metabolites were quantified using LCModel and water as internal reference.

#### Histological methods - brain

3 BDL rats and 3 shams were sacrificed at week 4 post-BDL for histological analysis. These rats did not undergo ^1^H-MRS scans or behavioural tests. Astrocytes were analysed by an anti-glial fibrillary acidic protein (GFAP) mouse monoclonal antibody (MAB360 Merck Millipore). Changes in water channel aquaporin 4 (AQP4) were followed using a rabbit polyclonal antibody (AB3594 Merck Millipore) as a marker of osmoregulation. Secondary antibodies were goat or rat anti-mouse or anti-rabbit IgG labeled with Alexa Fluor® 555 (red, Life Technologies). Immunostaining was performed as previously described^18^ (see supplementary materials).

#### Statistical methods

All results are presented as mean ± SD. One way ANOVA (Prism 5.03, Graphpad, La Jolla CA USA) with the Bonferroni’s multi-comparisons post-test were used to assess significance (*p < 0.05; **p < 0.01; ***p < 0.001; ****p < *0.0001*) in measured parameters. Pearson correlation analysis was performed on all longitudinally acquired data to test for correlations between measured parameters. Three types of correlations were performed: (1) to test overall correlation between changes during the whole progression of the disease (all longitudinally-acquired measurements between weeks 2 and 8); (2) to test correlations between changes only in the beginning of the disease (only values acquired at weeks 2 and 4 were used for the correlations); and (3) to test correlations between changes only at the end of the disease (values acquired at weeks 6 and 8 were used). Brain metabolites are expressed in absolute values (mmol/kg_ww_ (wet weight)) and in % difference between BDL rats and shams at each time-point in order to account for ongoing development.

More details on Methods can be found in the Supplementary data file.

## Results

### Validation of CLD and type C HE induced by BDL in P21 rats

#### Biochemical measurements

**Figure 1:**
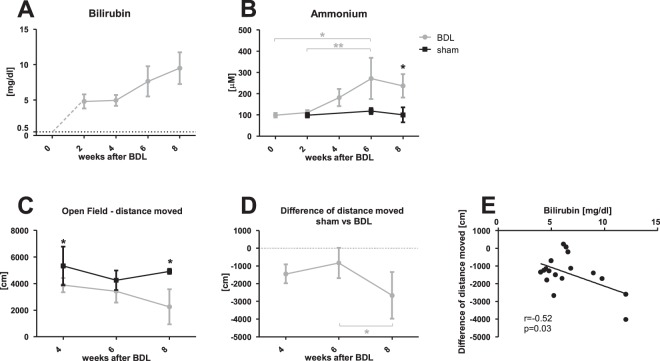
Blood parameters and behavioural tests. (**A**) Plasma bilirubin concentration in bile duct ligated (BDL) rats at each time-point. BDL rats before surgery and shams at all time points had plasma bilirubin levels under the detectable threshold of 0.5 mg/dl (dotted black line); plasma bilirubin levels in BDL rats were elevated from week 2 and rose through week 8. (**B**) Plasma NH4^+^ in BDL (grey line) and sham (black line) rats at each time point. NH4+ remained in physiological range in shams, and increased in BDL rats starting at week 4, reaching statistical significance at weeks 6 and 8 compared to week 0, 2 or shams. (**C**) Distance moved during Open Field test in sham (black) and BDL (grey) rats at each time point. (**D**) Difference in distance moved between BDL rats and shams at each time point. (**E**) Correlation between difference in distance moved and plasma bilirubin between weeks 4–8. *Comparison between shams and BDL at each time-point; *(grey) significance between time-points indicated on the graph.

Increased plasma bilirubin confirmed the presence of CLD in BDL rats: from undetectable in shams and before BDL (<0.5 mg/dl) compared to 9.5 ± 2.2 mg/dl at 8 weeks post-BDL (Data from different time points are summarized in Fig. 1A and Table S1). Plasma NH_4_^+^ averaged 103.9 ± 26.9 μM in shams throughout the study. BDL rats displayed a significant increase in plasma NH_4_^+^ at week 8 (237 ± 55 μM) (Fig. 1B and Table S1). In addition, BDL rats had measurably lower plasma glucose levels from week 4 (Fig. S1). Finally, BDL rats displayed significantly slower weight gain than sham operated animals from week 4 (Fig. S1).

#### Behavioural tests

Starting at 4 weeks post-BDL surgery, BDL rats showed significant decrease in the distance moved during the OF test compared to shams (Fig. 1C,D). Performance of BDL rats decreased further, reaching only 45% of shams at week 8. This decrease of motor activity in BDLs compared to shams correlated with plasma bilirubin levels (r = −0.52, p = 0.03, Fig. 1E). However no correlation was observed between deterioration in motor activity and increase in plasma NH_4_^+^. There was no difference between BDL rats and shams in latency to enter central zone or time spent in that zone (data not shown) at any time point, suggesting no difference in anxiety during the test between BDLs and shams.

### *In vivo*^1^H-MRS assessment of CNS metabolic alterations in a model of CLD acquired in pups

**Figure 2:**
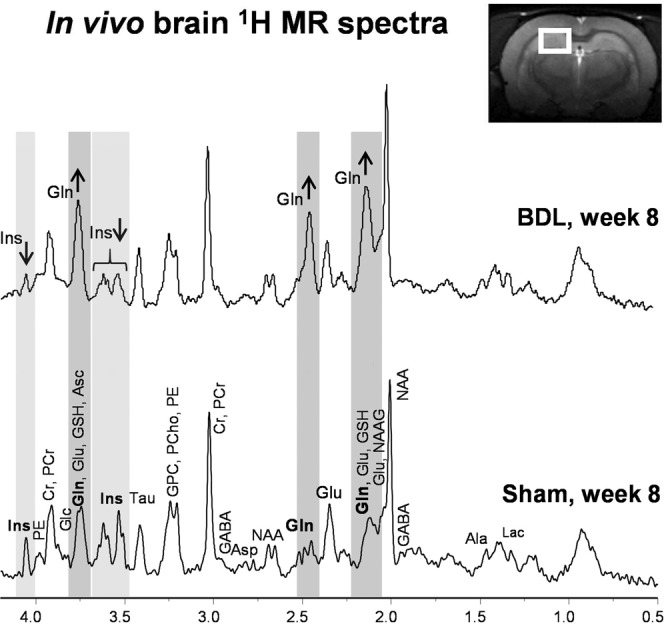
Representative *in vivo* 1H magnetic resonance (MR) spectrum from bile duct ligated (BDL) animal and sham animal. Spectra acquired 8 weeks after BDL or sham surgery, illustrating the quality of 1H-MRS data obtained. Visible increase in glutamine (Gln) in BDL rat compared to sham animal is highlighted in dark grey and the decrease in myo-Inositol (Ins) is highlighted in light grey. alanine (Ala), ascorbate (Asc), aspartate (Asp), glycerophosphocholine (GPC), phosphocholine (PCho), creatine (Cr), phosphocreatine (PCr), γ-aminobutyric acid (GABA), glucose (Glc), glutamate (Glu), glutathione (GSH), lactate (Lac), N-acetylaspartate (NAA), N-acetylaspartylglutamate (NAAG), phosphoethanolamine (PE) and taurine (Tau).

Spectra were characterized by a visible and quantifiable increase of Gln signal and decrease of mIns in the BDL rat 8 weeks post-BDL compared to sham (Fig. 2).

#### Gln and other main organic osmolytes

**Figure 3:**
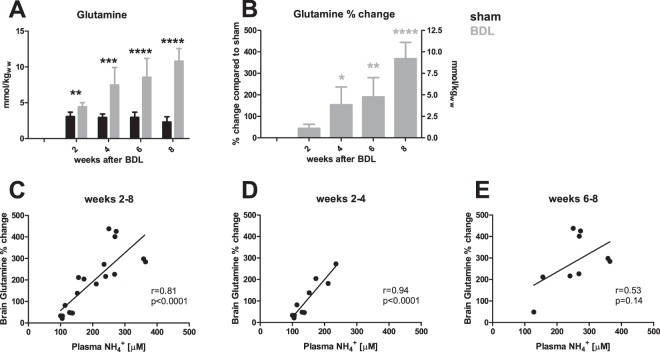
Brain glutamine and its correlations with plasma NH4^+^. (**A**) Time course of glutamine concentration in the hippocampus of sham (black) and bile duct ligated (BDL) (grey) rats, in absolute concentrations. (**B**) % difference in glutamine concentration between BDL rats and shams at each time point; left y-axis indicates % increase in glutamine and right y-axis expresses concentration in mmol/kgww (kg of wet weight) as compared to sham. (**C–E**) Correlations between plasma NH4^+^ and changes in brain glutamine during the course of the disease from weeks 2–8 (**C**), at disease onset between weeks 2–4 **(D**) and in advanced stages of biliary cirrhosis weeks 6–8 (**E**). *Comparison between shams and BDLs for each time-point; *(grey) significance compared to week 2.

One of the earliest observable metabolic changes in the hippocampus was an increase in Gln concentration 2 weeks post-BDL (+44%). It then increased progressively throughout the course of the study, reaching +368% at week 8 (Fig. 3A,B). Figure 3C illustrates the correlation between brain Gln and plasma NH_4_^+^ levels over the course of disease (week 2–8). Although the correlation was very strong in the early stages of the disease between weeks 2–4 (r = 0.94, p < 0.0001), it became non-significant between weeks 6–8 (r = 0.53, p = 0.14) (Fig. 3D,E).

**Figure 4:**
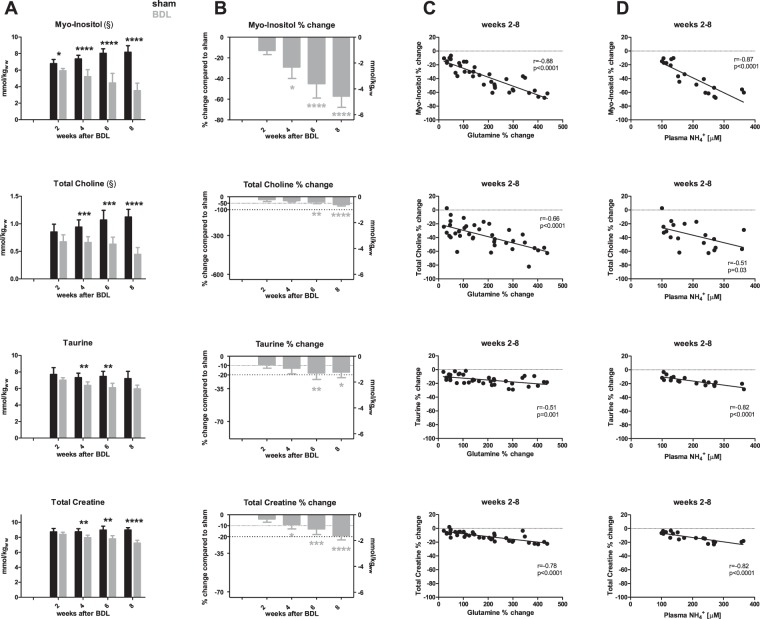
Brain organic osmolytes and their correlations with brain glutamine and plasma NH4^+^. (**A**) Time course of myo-inositol, total choline, taurine, total creatine in sham (black) and bile duct ligated (BDL) (grey) rats during disease progression expressed in absolute concentrations. (**B**) % difference in these metabolites between BDL rats and shams at each time point; left y-axis indicates % change of the metabolite and right y-axis represents corresponding mmol/kgww (kg of wet weight) change; the scale of the right y-axis is the same for all metabolites for ease of comparison of their contribution to the osmoregulation. (**C**) Correlations between changes in brain glutamine and changes in each individual metabolite between weeks 2–8. (**D**) Correlations between plasma NH4^+^ and changes in individual metabolites between weeks 2–8. *Comparison between shams and BDL for each time-point; *(grey) significant difference compared to week 2; (^§^) indicates a significant change in sham animals as a function of age.

Gln increase was mirrored by a decrease in mIns, which was significant from week 2 (−13%) and reaching −57% at week 8 (Fig. 4A,B). Other major brain organic osmolytes displayed a comparative delay in their decrease, reaching significance 4 weeks post-BDL. Maximal changes in concentration reached −60% for tCho, −17% for Tau, −19% for total creatine (tCr) at 8 weeks post-BDL. Correlations between these metabolites (mIns, tCho, Tau or tCr) and brain Gln or plasma NH_4_^+^ are presented in Fig. 4C,D.

#### Energy metabolites and metabolic stress: creatine, phosphocreatine and lactate

**Figure 5:**
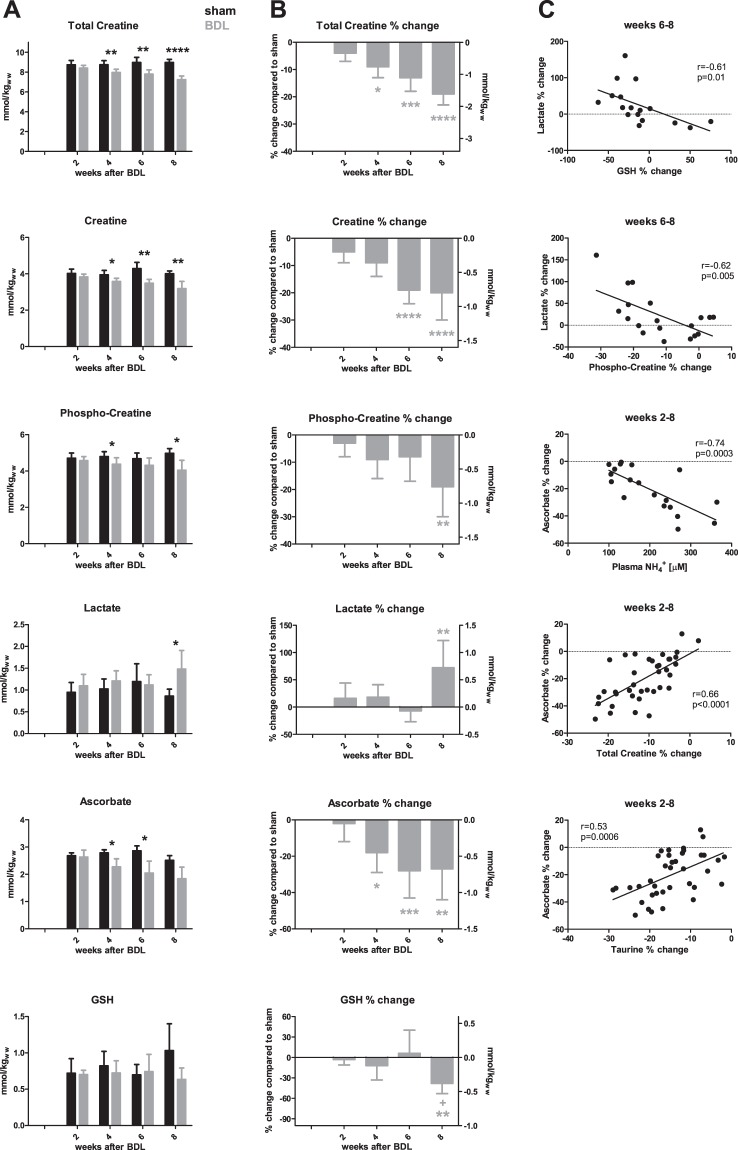
Brain energy metabolites and antioxidants and their correlations. (**A**) Brain metabolites in shams (black) and bile duct ligated (BDL) (grey) rats during the progression of the disease, expressed in absolute concentrations. (**B**) % difference in metabolite concentration between BDL rats and shams at each time point. Left y-axis: % change of the metabolite, right y-axis represents corresponding mmol/kgww (kg of wet weight) change. (**C**) Correlations of lactate and ascorbate. *Comparison between shams and BDL for each time-point; *(grey) significance compared to week 2; + significance compared to week 4.

Discussed above as an osmolyte but essentially known for its role in energy metabolism, tCr decreased gradually during disease progression, reaching a significant −9% decrease at week 4 and progressing to −19% at week 8 (Fig. 5A,B). This change correlated with the increase in Gln (r = −0.78, p < 0.0001) (Fig. 5C). Both creatine (Cr) and phosphocreatine (PCr) were significantly less concentrated in the hippocampus of BDL rats compared to shams at 4 weeks post-BDL. At week 8 Cr showed a decrease of −20%, similar to PCr (−19%).

An increase in lactate (Lac) was present only at the very end of the disease (week 8; Fig. 5A,B). The significant increase of +70% at week 8 correlated between weeks 6–8 with the decrease in glutathione (GSH) (r = −0.61, p = 0.01) and PCr (r = −0.62, p = 0.005), but not with Cr (Fig. 5C).

#### Antioxidants: Ascorbate and GSH

Our *in vivo* measurements also showed a decrease in antioxidant concentrations in the hippocampus of the BDL rats. Ascorbate (Asc) decreased gradually during the course of disease (decrease of −18% at week 4 continuing to −27% at week 8; Fig. 5A,B). Its decrease was correlated with plasma NH_4_^+^ levels (r = −0.74, p = 0.0003), and this correlation was stronger than with Gln (r = −0.44, p = 0.01) (Fig. 5C). There was no correlation with plasma bilirubin. The correlation with tCr (r = 0.66, p < 0.0001) was stronger between weeks 2–4 (r = 0.81, p < 0.0001) than between weeks 6–8 (r = 0.31, p = 0.19); the same was true for Tau: correlations between weeks 2–8 (r = 0.53, p = 0.0006), between weeks 2–4 (r = 0.62, p = 0.005) and weeks 6–8 (r = 0.23, p = 0.34) (data not shown). On the other hand, hippocampal GSH concentrations remained constant until week 6, decreasing significantly (−38%) at week 8 (Fig. 5A,B).

#### Neurotransmitters: Glutamate, aspartate and GABA

**Figure 6:**
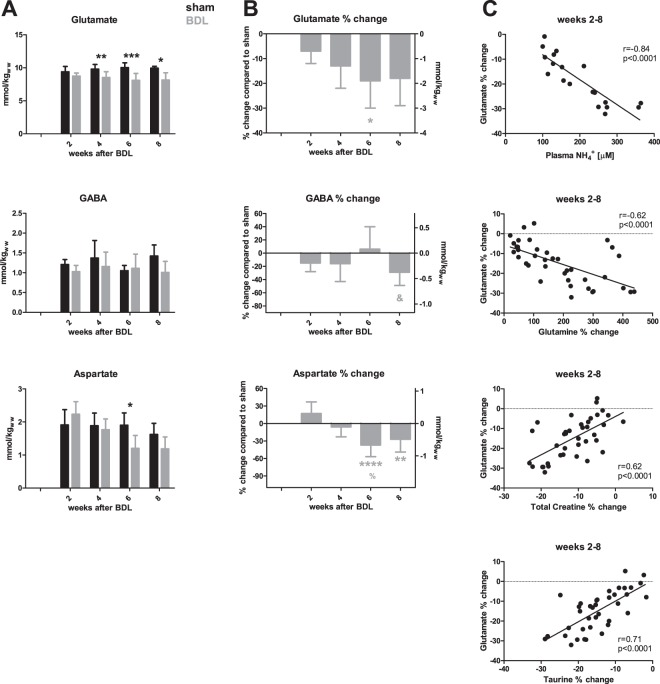
Neurotransmitters and their correlations. (**A**) Changes in brain metabolite concentrations in sham (black) and bile duct ligated (BDL) (grey) rats throughout the study, in absolute concentrations. (**B**) % difference in metabolites between BDL rats and shams at each time point. Left y-axis: % change. Right y-axis: change in mmol/kgww (kg of wet weight). (**C**) Correlations of glutamate with blood and brain measurements. *Comparison between shams and BDL for each time-point; *(grey) significance compared to week 2; % indicates significance compared to week 4; & significance compared to week 6.

We observed a decrease in all measurable neurotransmitters (Fig. 6A,B). Glutamate (Glu) was significantly less concentrated in the hippocampus of BDL compared to shams from week 4 (−13%) and further decreased at week 8 (−18%). The most significant correlations of Glu are presented in Fig. 6C. Aspartate (Asp) decreased significantly by −37% at week 6 and was at −27% at week 8, compared to shams. Finally, γ-aminobutyric acid (GABA) showed a significant −29% decrease at week 8. Changes in GABA concentrations correlated with GSH (r = 0.83, p < 0.0001), Tau (r = 0.50, p = 0.03) and tCr (r = 0.57, p = 0.01) between weeks 6–8 (data not shown).

#### Metabolic changes during brain development

It is well known that brain metabolite concentrations change during brain development until P28^24,25^. Given that ^1^H-MRS measurements in this study were performed on rats between P35–P77, we evaluated possible metabolic changes in the hippocampus of sham BDL rats. The only metabolites that showed significant change in shams during this period were mIns and tCho (Fig. 4A).

#### Astrocyte changes and dysregulation of AQP4 in BDL rat pups

**Figure 7:**
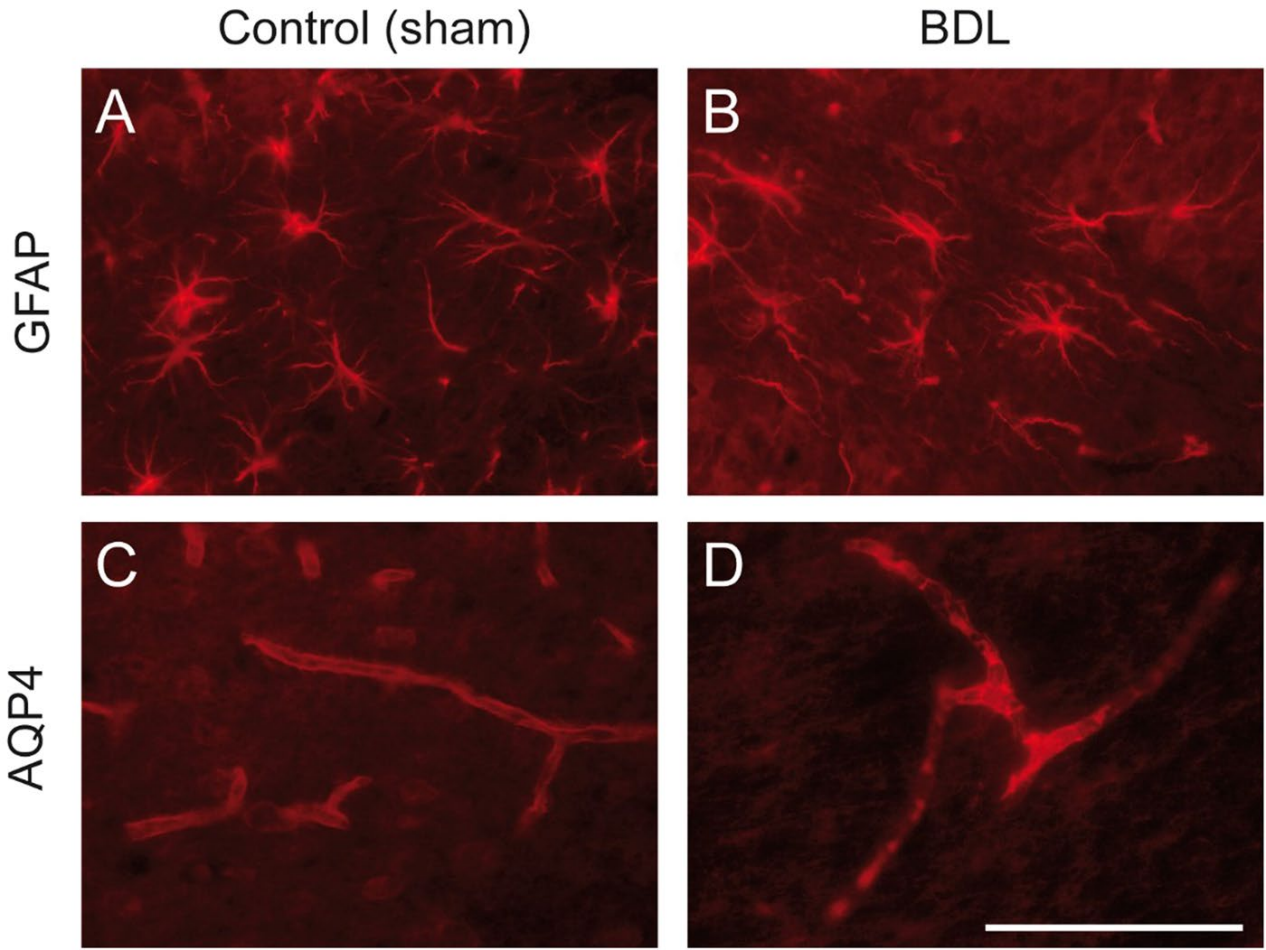
Astrocytic dysregulation in bile duct ligated (BDL) rat pups: glial fibrillary acidic protein (GFAP) (**A,B**) and aquaporin 4 (AQP4) (**C,D**) expression in hippocampus (hilus) 4 weeks post-BDL: BDL (**B,D**) versus sham (**A,C**). (**A,B**) BDL induced increased GFAP expression in astrocytes, and a thickening of proximal, versus retraction of distal, astrocytic processes compared to normally differentiated astrocytes in sham-operated animals. (**C,D**) BDL led to increased AQP4 expression at the blood-brain barrier in both the astrocytic feet lining microcapillaries and microcapillary endothelial cells. Representative pictures taken from BDL and control sham rats.

Using GFAP staining, we demonstrated that BDL rat pups, compared to sham-operated controls, developed time-dependent changes in astrocyte morphology, already observable at 4 weeks post-BDL (Fig. 7). Astrocytes showed an increase of GFAP expression and the thickening of their main proximal processes. A decrease in numbers and the retraction of their distal processes were also observed (Fig. 7A,B). In healthy rats, AQP4 is expressed both in astrocytic feet lining microcapillaries and within microcapillary endothelial cells at the BBB. In the young BDL rats AQP4 expression was increased in the hippocampus as soon as 4 weeks post-BDL (Fig. 7C,D).

Additional results including liver histology can be found in the Supplementary data file (Figs. S1–S4).

## Discussion

This first detailed description of the longitudinal neurometabolic changes induced by experimental biliary cirrhosis in a young rat model, shows that rats having developed cholestatic liver disease as pups display previously unknown molecular changes in addition to the characteristic findings of HE described in adult rats and human subjects^10–14^. In addition, the kinetics of some of the neurometabolic changes are different than those observed in rats having undergone BDL as adults. Taken together these findings suggest that the developing brain displays a unique vulnerability to chronic cholestatic liver disease, possibly offering insight into the long-term neurocognitive changes observed in patients having developed biliary cirrhosis in childhood.

The neurometabolic profile in the hippocampus of rats with experimental biliary cirrhosis induced by BDL at P21 was characterized by a progressive rise in Gln which correlated tightly with plasma NH_4_^+^ during the first four weeks of an 8-week longitudinal observation period. Osmoregulatory decrease of mIns preceded that of Tau, tCho and tCr. Further, a progressive decrease in Asc and Glu correlated negatively with plasma NH_4_^+^ concentration. There was a very progressive decrease in PCr throughout the study, which culminated at 8 weeks, concurrent with an increase in Lac and a decrease in GSH concentrations. These observations were probably related to the metabolic consequences of end-stage liver disease, but also suggested brain energy metabolism perturbation, an area still incompletely understood.

### Neurometabolic response to plasma ammonium

Liver disease is characterized by an increase in peripheral NH_4_^+^ load which reaches the CNS, where the only detoxifying pathway is the production of Gln by glutamine synthetase (GS) in astrocytes. Eight weeks post-BDL surgery, young BDL rats experienced a 4.5-times increase of Gln in hippocampus, compared to 2.4-times in adult BDL rats^18^ (Figs. S2 and S3). Plasma NH_4_^+^ and bilirubin levels also reached higher values in young vs adult BDL rats. 8 weeks post-BDL, plasma NH_4_^+^ was 75% higher in pups than in adults and plasma bilirubin was 41% higher (Fig. S2), suggesting that rats ligated as pups may be sicker than their adult counterparts, and therefore that variations in neurometabolic profile between pups and adults may be secondary to the severity of liver disease. While disease severity might contribute to higher brain Gln, we suggest that the difference in brain Gln between young and adult BDL rat may be also due to difference in the effect of NH_4_^+^ on maturing metabolic processes. Not only was the correlation between plasma NH_4_^+^ and brain Gln stronger in the young animals (r = 0.81, p < 0.0001) than in adults (r = 0.61, p = 0.004), but the slope of the linear increase between plasma NH_4_^+^ and hippocampal Gln was twice as strong in young animals than adults (Fig. S2). Whether this was due to GS kinetics in the developing brain or BBB maturation are pending questions.

The tight correlation between Gln and NH_4_^+^ held true until week 4 post-BDL, but was lost thereafter (Fig. 3C–E). This was likely due to fact that brain Gln continued to rise between weeks 6 to 8 post-BDL, whereas plasma NH_4_^+^ reached a plateau. This biological decoupling of two tissues is probably explained by different NH_4_^+^ concentrations between blood and CSF/brain. Interestingly, plasma bilirubin and brain Gln were also correlated in this model. Whether there is a genuine link between these two parameters beyond disease severity remains to be determined. Similarly, the negative correlation of distance moved with plasma bilirubin may be interpreted in one of two ways: either this finding points to bilirubin as a neurotoxin or circulating bilirubin levels serve as a surrogate for severity of liver disease.

NH_4_^+^ impacts the CNS both through direct toxicity and indirect metabolic pathways^26,27^, something which we have previously shown in BDL rats^18^. This finding is further supported by studies showing that HE disappeared when GS was blocked experimentally during chronic hyperammonemia^28,29^. In the present study, several neurometabolic changes correlated very strongly with plasma NH_4_^+^. Whether this is due to a direct effect of NH_4_^+^ on a young brain or to a rapid succession of molecular events in the CNS downstream of elevated plasma NH_4_^+^ remains to be determined.

### Response to osmotic stress

As expected, we observed an increase in hippocampal Gln concentration and a decrease in mIns and tCho. Pups displayed the first significant alterations in Gln and mIns at week 2 post-ligation while in adult BDL rats the increase in Gln was significant only from week 4 post-BDL and the mIns decrease from week 6 (Fig. S3).

The early and very profound decrease of mIns (almost −60% at week 8 compared to only −30% in adult BDL rats^18^) in BDL rats having undergone BDL at P21, may have far reaching implications considering the pleiotropic role of mIns in neurometabolism and development. mIns is central to phospholipid metabolism, membrane formation and intracellular signaling during brain growth^30^. The significance of the observed decrease in the present model is highlighted by the physiological increase of mIns in the hippocampus of sham rats during this developmental window.

The observed decrease in tCho may also be interpreted as an intracellular osmoregulatory response. Choline is required for membrane phospholipid synthesis and myelination^31^, and as a precursor of acetylcholine is essential for cognition and for normal memory development^32^. Therefore, in the context of brain development, the observed −60% decrease 8 weeks post-BDL in the hippocampus of young rats, compared to only −24% in hippocampus of adult BDL rats^18^ may have far-reaching neurocognitive consequences (Fig. S3).

Likewise, Tau decreased sooner in rats with CLD acquired as pups than as adults (4 weeks post-BDL compared to 6 weeks in adults) and the absolute decrease at week 8 was −17% compared to −10% in adults^18^. In the CNS, Tau is present in both glial cells and neurons^33^. Therefore, it is not clear whether its decrease is linked to astrocytic osmoregulation or to neuronal alterations. In the present study, Tau was the only osmolyte correlating more significantly with plasma NH_4_^+^ than with brain Gln. Further, its concentration decreased 2 weeks after the mIns decrease, known to occur in astrocytes, suggesting that in HE Tau may play a role beyond osmoregulation in astrocytes. Given the antioxidant properties of Tau, this relative Tau deficiency may further contribute to cellular stress^34^, adding to the list of metabolically important molecules that are significantly decreased in the hippocampus of rats having acquired CLD early in life.

Despite molecular evidence of ongoing osmoregulation, astrocytes displayed a decreased number and shortening of processes, and a thickening of proximal processes at 4 weeks post-BDL, akin to what was observed in adult rats^18^. This was paralleled by an increase of AQP4 expression, a transmembrane channel regulating water homeostasis^35^, normally expressed in the astrocytic feet and in microcapillary endothelial cells around the BBB, as previously shown in culture and experimental animals^36^. This suggests that BDL-induced osmotic stress and increased AQP4 may contribute to morphological changes in astrocytes, something recently shown in adult BDL rats^18^. The pathophysiology and functional implications of the observed changes in astrocyte morphology are still incompletely understood, given that these changes have also been shown in hyperammonemic rats without CLD and may differ according to brain region^37–40^. Although the neurometabolism of rats having undergone BDL at day 21 differs from that of adults, our data suggest that the morphological changes are rather similar^18^.

Interestingly we show that GFAP is increased in the hippocampus of pups at 4 weeks post BDL. This may appear in contradiction with previous work showing decreased GFAP in type C HE, particularly in adults and in late stages of the disease^41^. However, one might rather look at these results as a continuum. Indeed, we recently showed that there is an increase of GFAP signal at 4 weeks post BDL in adult rats, followed by a decrease at the late stages of the disease (8 weeks post BDL)^18^. Taken together, our findings suggest that both in the pup (this study) and in adult BDL models^18^, astrocytes may first be highly reactive as illustrated by increased GFAP, increased size of proximal processes, and decreased number and retraction of distal processes. We argue that this phase likely precedes the characteristic decrease in GFAP, suggestive of major cellular changes associated with advanced disease, but these kinetics warrant further exploration.

### Energy metabolism

Cr, PCr and tCr were significantly decreased in experimental animals compared to sham-operated animals 4 weeks post-BDL. The observed decrease in tCr of −20% 8 weeks post- BDL was greater than that observed in adult BDL rats (−8%)^18^. The significance of this finding lies in the documented association between a 15% decrease in tCr and impairment of axonal growth in organotypic brain cell cultures in development exposed to NH4+^6^,^42^. In our in vivo experiments, we observed a decrease of Cr starting early in the disease. While osmotic regulation may contribute to this decrease, an inhibition of brain Cr synthesis may also be involved, in particular at later stages of the disease, as arginine:glycine amidinotransferase (AGAT), the first of the two enzymes involved in Cr synthesis, is inhibited in organotypic brain cell cultures in development exposed to NH4+^42^. The 20% deficit in Cr observed over 8 weeks may thus be reached through a combination of the natural degradation of Cr (nearing 2% per day), an inhibition of brain Cr synthesis, and osmotic regulation. Regardless of the underlying mechanism, this decrease in tCr at a time of axonal growth may seriously impact brain development and induce energy metabolism disturbances.

Lac concentration peaked at week 8, something not observed in adult BDL rats (Fig. S3). Although this may reflect ongoing energy dysfunction, its increase more likely reflects the intense metabolic, inflammatory and oxidative stress at the end of the disease course. The most likely interpretation is that this Lac peak is the signature of a terminal, neurometabolic storm rather than a novel molecular mechanism in HE. However, more detailed investigation of energy metabolism in this developing brain model of type C HE is warranted.

### Response to oxidative stress

Oxidative stress is known to play a role in type C HE pathophysiology. Asc and GSH are the most concentrated non-enzymatic antioxidants in the brain. They have slightly different but complementary roles. Asc is localized preferentially in neurons and GSH in glial cells^43^. In our model, Asc concentration decreased first, and was significantly reduced from week 4 post-BDL. The observed decrease correlated with plasma NH_4_^+^ elevation, suggesting a direct toxicity via NH_4_^+^- induced reactive oxygen species production^44,45^. As liver disease progresses, decreased synthesis by the liver may contribute to declining Asc concentrations in the brain, but this remains to be studied in detail. The previously described decrease in Tau and tCr concentrations, two molecules also known for their antioxidant properties, correlated with Asc decrease, suggesting that they may also contribute to the response to oxidative stress in this model^34,46^.

The role of Asc in the brain extends beyond its antioxidant properties to include neuroprotective and neuromodulatory functions, for example against Glu^47,48^, all of which could be significantly impacted by a −30% decrease at week 8. Akin to all the other quantified metabolites, changes in Asc concentration also displayed a significantly stronger decrease in young vs adult BDL rats (−13% decrease at week 8^18^). In contrast to Asc, GSH decreased at week 8 post-BDL in young rats, something not observed in adult rats^18^. GSH synthesis is dependent on precursor availability: cysteine and Glu^49^. Therefore, in advanced stages of HE, its synthesis could be impaired due to lack of available Glu. It is also known that GSH is consumed in mitochondrial dysfunction and metabolic stress such as might be observed in end-stage liver disease at week 8. Elevated Lac concentrations in the hippocampus of young rats and its correlation with GSH between weeks 6–8 suggests that this mechanism might be involved in our model.

### Neurotransmitters

The experimental advantages of high field ^1^H-MRS and high quality shimming afforded us the opportunity to separate Gln and Glu resonances in the spectrum and to measure their concentrations individually. The decrease in Glu appeared slightly earlier in young BDL rats than in adults (4 weeks post-BDL compared to 6 weeks in adults), and at 8 weeks post-BDL the overall change was −18% in young BDL compared to −13% in adults^18^ (Fig. S3). Decreased Glu concentration in HE may occur for several reasons. One possibility is its increased use for astrocytic Gln synthesis from NH_4_^+^. Unlike in adult BDL rats, the correlation of plasma NH_4_^+^ with brain Glu was stronger than that between brain Gln and Glu, suggesting a direct effect of plasma NH_4_^+^ on Glu decrease. This may be secondary to an immature glutamatergic system in the developing brain, or changes in glutamate transporter function or number. Finally, the correlation of Glu decrease with Tau and tCr suggests its consumption may also be related to energy metabolism or neuroinflammation^50^. The most likely, however, is that the origin of the observed decrease in Glu hippocampal concentration is multifactorial.

The decrease in hippocampal GABA concentration observed over the course of the study seems to be a specific feature of the developing brain under BDL conditions, as no such change was observed in adult BDL rats^18^. These findings need to be interpreted with caution for two reasons. First, this decrease was observed at the very late stages of the disease, possibly reflecting terminal changes. Second, this may be secondary to the significant decrease in Glu, the main GABA precursor, observed in pups. The aforementioned Tau decrease may also play a role in the regulation of GABAergic neurotransmission, given that it is a GABA_A_ receptor agonist^51^. These *in vivo* findings are in keeping with changes in GABAergic neurotransmission changes reported in the adult brain with type C HE^52,53^. However, they contrast with reports in developing brain cell aggregates which displayed no change in GABA-ergic neurotransmission under NH_4_^+^ treatment^6^. How to reconcile these findings deserves further *in vivo* studies in young animals.

To conclude, we demonstrated for the first time longitudinal changes in a model of CLD and type C HE in young rats during late rat brain development between post-natal days 21 to 77 (corresponding to human brain development from 8 months- 8 years) and compared them to adult rats with the same disease. Using *in vivo*, longitudinal measurements of more than 15 brain metabolites, we observed a rapid increase in hippocampal Gln correlated with plasma NH_4_^+^. This was followed by a decrease in other osmolytes, with evidence of oxidative and metabolic stress, as well as perturbations in neurotransmitters and, importantly, a decrease in tCr. Neurometabolic changes were accompanied by changes in astrocyte morphology and increased AQP4 expression, observed already 4 weeks post-BDL and in keeping with findings in adult BDL animal.

Taken together, CLD-induced HE in the developing brain appears to be a multifactorial disease akin to what is accepted in adult models. Many of the changes are similar in animals having undergone BDL as pups or adults. There were however several striking differences: (1) all neurometabolic changes were more pronounced in the developing brain, (2) some changes appeared earlier (Gln, osmolytes mIns, tCho, neurotransmitter Glu) (3) significant changes in Asp, GABA, Lac, PCr, and GSH appeared to be unique to the young brain. Furthermore, most of the metabolic changes in the developing brain correlated better with rising plasma NH_4_^+^ than with increasing brain Gln, suggesting a direct effect of NH_4_^+^ on metabolism.

Therefore, we conclude that the developing hippocampus does indeed display increased vulnerability to the metabolic insults of CLD compared to what is observed in adult animals. Whether this paradigm extends to other brain areas, how they impact short-term and long-term function of these respective areas, and whether the developing brain is equally vulnerable during other developmental windows remains to be elucidated.

## Supplementary information

Supplemntary materials.

## Data Availability

The datasets generated during and/or analysed during the current study are available from the corresponding author on reasonable request.
