# Supplementary material for: Longitudinal osmotic and neurometabolic changes in young rats with chronic cholestatic liver disease
Source: Sci Rep. 2020 May 5;10:7536. doi: 10.1038/s41598-020-64416-3 (PMC7200786; doi:10.1038/s41598-020-64416-3)
Supplement: Supplementary file 1 — Supplemntary materials. [file 41598_2020_64416_MOESM1_ESM.doc]

SUPPLEMENTARY MATERIAL

**Title: Longitudinal osmotic and neurometabolic changes in young rats with chronic cholestatic liver disease**

**Authors:** Veronika Rackayova^1^, Olivier Braissant^2^, Anne-Laure Rougemont^3,4^, Cristina Cudalbu^5^, Valérie A. McLin^4, 6*^

**Methods**

*Study design*

Twelve (12) male Wistar pups underwent bile duct ligation (BDL) and 7 animals were sham operated on postnatal day 21 (P21). Blood sampling was obtained from the sublingual vein at the beginning of the afternoon. *In vivo* brain ^1^H magnetic resonance spectroscopy (MRS) scans were performed at week 2, 4, 6 and 8 post-BDL. Behavioural tests were performed to measure motor activity at week 4, 6 and 8 post-BDL, before MRS scans. For ^1^H-MRS, 12 BDL and 7 shams were scanned at each time-point, except for week 2 were 7 BDL rats were scanned and week 8 were 7 BDL and 5 sham rats were scanned. Lower number of BDL rats at week 2 and sham rats at week 8 is due to limited scanning time. Also, 5 BDL rats were sacrificed between weeks 6 and 8 for the welfare of the animals. Blood sampling and behavioural tests were performed on smaller number of animals (5 BDL and 4 shams for blood sampling; 7 BDL and 5 shams for behavioural tests). Animals were kept under 12h/12h light/dark cycle with water and food *ad libitum*. All the experiments were performed during light cycle.

We used a previously published neuroinformatics approach [20] to approximate rat- and human- brain development. Although brain development is remarkably conserved across mammals [21], making this comparison feasible, each species develops at its own rate. Further, brain regions themselves mature at a variable pace. For the purposes of this study, myelination, neurogenesis, axonal growth of the limbic system of the P21 rat (day of BDL surgery) is estimated to be the equivalent of an 8.5 moth old human while P77 old rats (end of study: week 8 post-BDL or post coital age 98 days) approximate an 8.5 year old human (<http://translatingtime.org/translate>).

*Histological methods - brain*

At the time of sacrifice (by decapitation, handled according to the rules of the Swiss Academy for Medical Sciences), brains were immediately dissected and rinsed in ice-cold phosphate buffer saline (PBS) followed by fixation. Brains were fixed in chilled 4% paraformaldehyde in PBS (PFA-PBS; Sigma-Aldrich, Germany) overnight at 4˚C. Immunohistochemistry was performed on brain tissue as previously described [19]. Briefly, following overnight fixation in PFA-PBS, brains were sequentially infiltrated with 12% and 18% sucrose (Calbiochem, Germany) in PBS at 4˚C. The brains were then washed with PBS to remove the external excess of sucrose and embedded in O.C.T compound (Tissue-Tek, Netherland), frozen in liquid nitrogen-cooled isopentane (Sigma-Aldrich, Germany) and stored at -80˚C until used for cryosections. For immunohistochemistry, 16 μm cryosections of brain tissue were prepared (LEICA Frigocut 1850; Germany) and fixed 15 min in PFA-PBS at room temperature. After 3x 5 min washes in PBS, non-specific antibody binding sites were blocked 1h at room temperature with 1% bovine serum albumin (BSA; Sigma-Aldrich) in PBS (BSA-PBS). Primary anti-GFAP and anti-AQP4 antibodies diluted 1:200 in 1% BSA-PBS where applied to sections O/N at 4°C, then detected with fluorescent secondary antibodies (1:200, 2h RT) labelled with Alexa Fluor® 555. Sections were mounted under FluorSave Reagent (Calbiochem) and observed using an Olympus BX50 microscope equipped with appropriate filter for red fluorescence. Stained sections were photographed with a UC30 digital camera mounted on the microscope, allowing image processing with the Cell Sens Imaging Software (Olympus). Analysis of immunostaining intensity and of the changes in astrocyte morphology was performed as described in details in our recent publication [19].

*Histological methods- liver*

At the time of sacrifice (by decapitation, handled according to the rules of the Swiss Academy for Medical Sciences), livers were immediately dissected and rinsed in ice-cold PBS followed by fixation in 4% paraformaldehyde (PFA). They then underwent gross processing and paraffin-embedding. Hematoxylin & Eosin (H&E) staining was performed on 3µm thick sections of the paraffin-embedded tissue. Sirius Red was used for collagen assessment in 3 BDL and in 3 sham rats.

**Results**

Table S1 – Evolution of plasma ammonium and bilirubin levels in rats that underwent BDL surgery at post-natal day 21 (week 0).

|  | ammonium [μM] | p-value | bilirubin [mg/dl] | p-value |
| --- | --- | --- | --- | --- |
| week 0 | 98.5±10.6 | ns | <0.5 |  |
| week 2 | 111±11.7 | ns | 4.8±1.0 |  |
| week 4 | 181.6±40.8 | ns | 5±0.8 | ns |
| week 6 | 271.6±97 | * vs week 0  ** vs week 2 | 7.6±2.1 | ** vs week 2  ** vs week 4 |
| week 8 | 237±54.9 | * vs shams | 9.5±2.2 | **** vs week 2  **** vs week 4 |

*Figure S1*

*Figure Legend S1:*

*(A): Growth curve of bile duct ligated (BDL) and sham animals between post-natal days 21 (week 0) and 77 (week 8). (B): Plasma glucose in BDL and sham animals. Blood sampling was performed at variable times during the day, probably contributing to large variations in plasma glucose.* (** p < 0.05; ** p < 0.01; *** p < 0.001; **** p < 0.0001*)

*Figure S2*

*Figure Legend S2:*

*(A,B): Difference in the evolution of plasma bilirubin and ammonium in pup (grey) and adult (black) bile duct ligated (BDL) rats. (C): Correlation of plasma ammonium concentration and change in brain glutamine pup (grey) and adult (black) BDL rats.* (** p < 0.05; ** p < 0.01; *** p < 0.001; **** p < 0.0001*)

*Figure S3:*


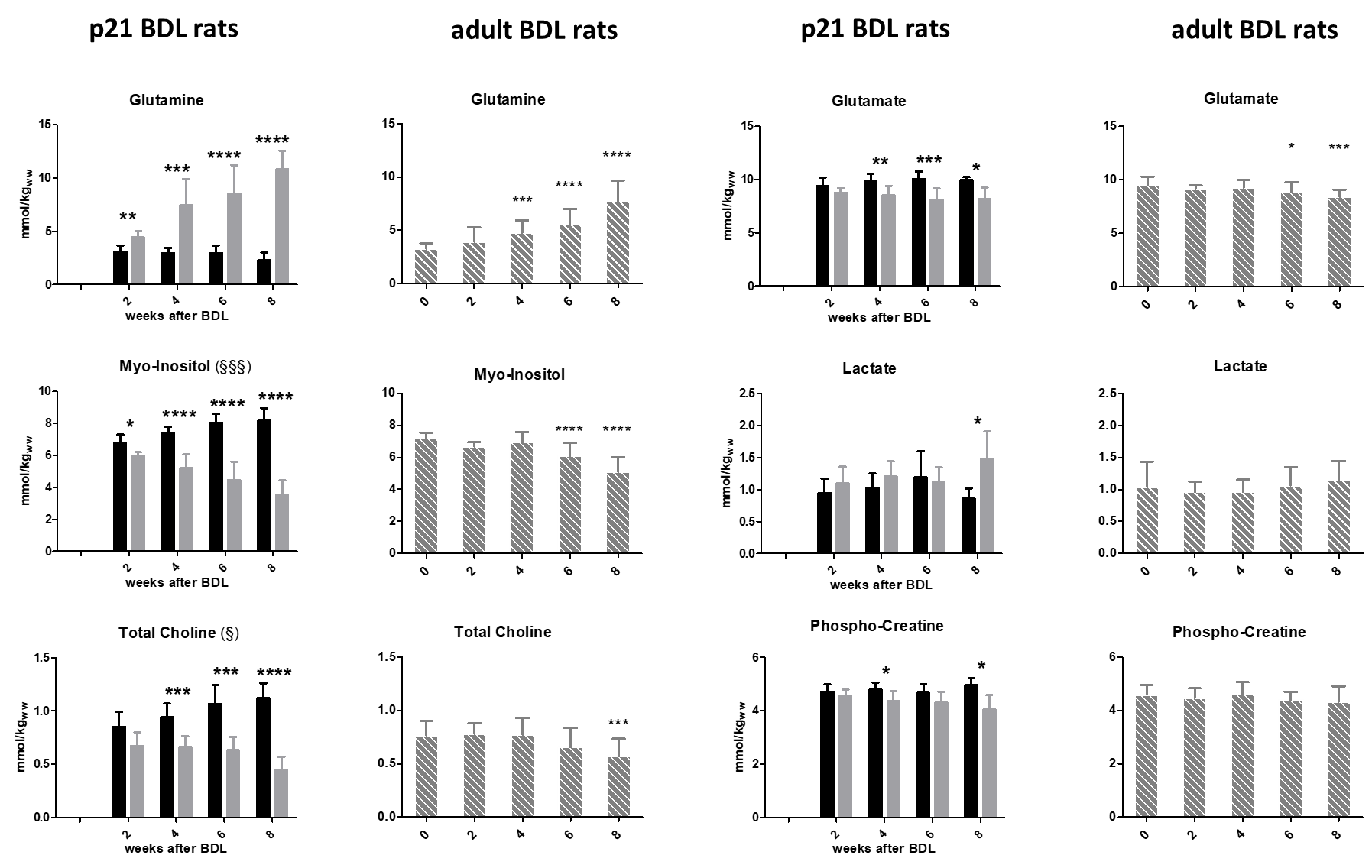


*Figure Legend S3:*

*Differences in the time-course of brain metabolites in pup- (black and grey) and adult- (grey lines) bile duct ligated (BDL) rats. The adult BDL data were previously published in* [19].

*Stable metabolites during CLD: We did not measure any changes in the concentrations of the following during the course of the study: Ala, PE, NAA, NAAG and tNAA.* (** p < 0.05; ** p < 0.01; *** p < 0.001; **** p < 0.0001*)

*Liver - gross and histological findings*

*Sham animals*

Upon gross evaluation, livers from sham animals were unremarkable, and histology displayed only mild and rather non-specific findings, such as vascular congestion. Rare and minute lobular lymphoid cell aggregates were seen. No relevant portal tract fibrosis was seen (Fig. S4A) and the portal tracts showed a centrally-located interlobular bile duct, and no bile duct proliferation (Fig. S4B).

*Animals with bile duct ligation*

Grossly, the livers after bile duct ligation were micronodular or slightly nodular. Upon histological evaluation, bile duct proliferation was seen in all the livers, expanding the portal tracts. Interlobular bile ducts were frequently dilated and angulated, lined by an attenuated epithelium. Accompanying fibrous bands were seen, bridging the portal tracts (Fig. S4C and S4D), the reticulin stain highlighting the increased fibres, surrounding and, in one of the three livers, disrupting the nodules (Fig. S4E). In the same liver, intermediate structures were made of hepatocytes arranged in a pseudoacinar pattern with a central developing lumen (Fig. S4F). Apoptotic bodies were seen in the portal tracts and in the nodules, and a few mitoses were observed both in cholangiocytes and in hepatocytes. Inflammation and cholestasis were only minimal, as was steatosis.

*Figure S4*


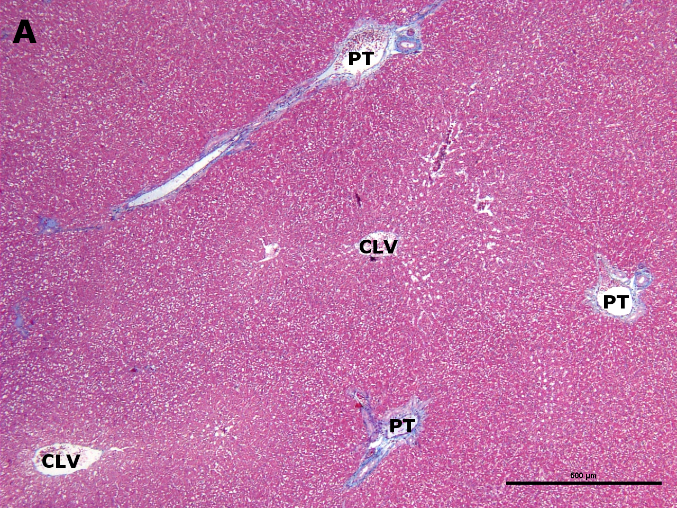

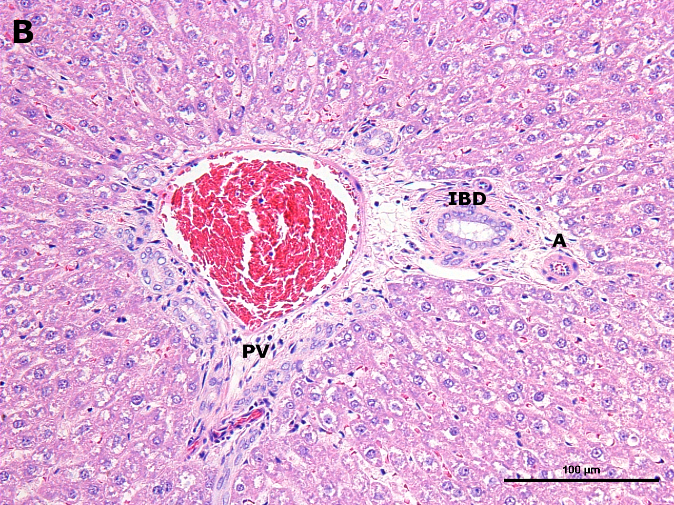

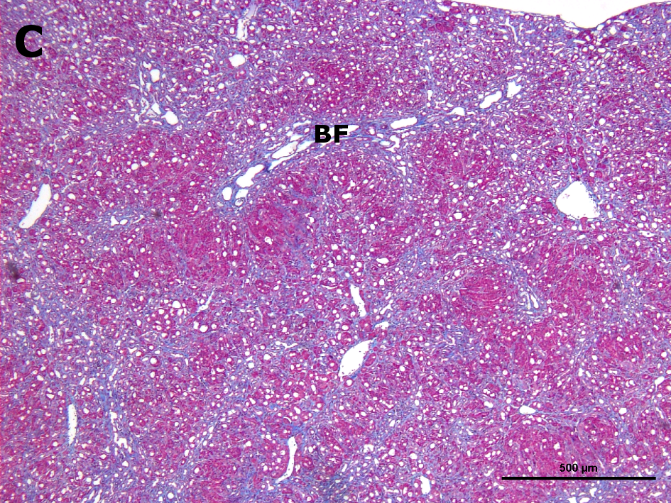

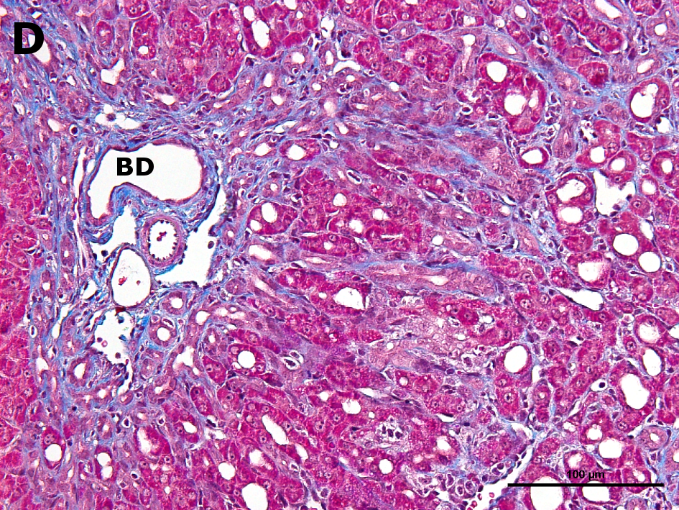

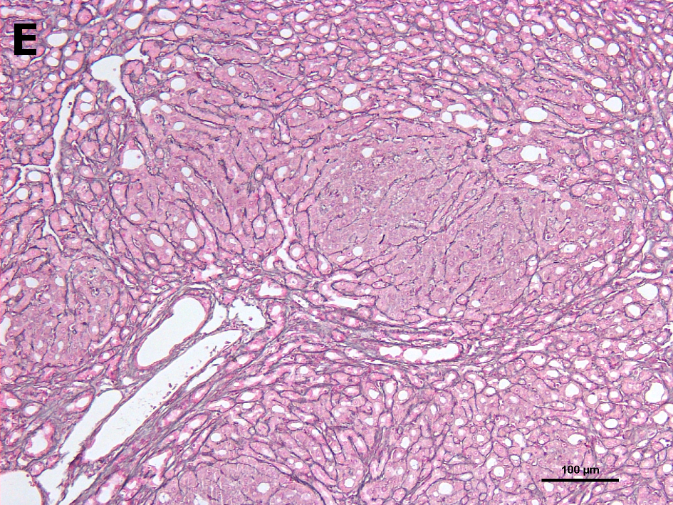

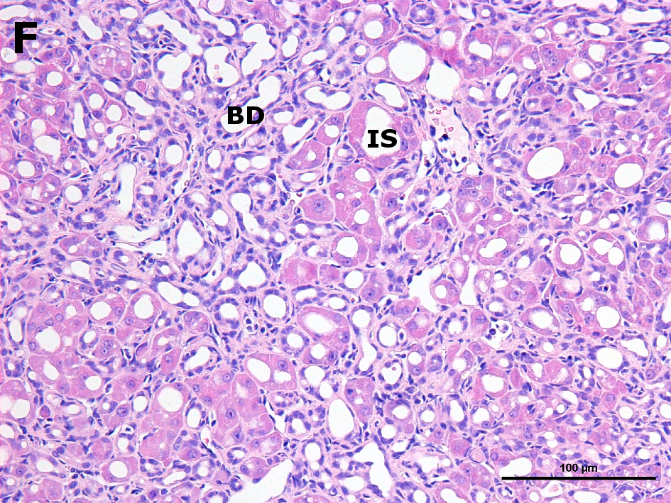


*Figure Legend S4.*

*Representative histological findings in sham animals and after bile duct ligation (BDL).*

*(A,B): Sham p21 pup. (A): Portal tracts (PT) and centrilobular veins (CLV) are evenly distributed and show no fibrosis (Masson's trichrome stain, original magnification x40). (B): At higher magnification, portal tracts show an interlobular bile duct (IBD), and few peripheral ductules, a portal vein (PV) and a segment of the hepatic artery (A) (Hematoxylin&Eosin, H&E x200).*

*(C-F): BDL p21 pup, showing extensive fibrosis, and architectural changes. (C): Fibrous tracts bridge portal tracts and centrolobular veins (BF), disrupting the lobular architecture (Masson's trichrome stain, x40). (D): Portal tracts are expanded and show a proliferation of dilated and irregular bile ducts (BD), lined by attenuated cholangiocytes (Masson's trichrome stain, x200). (E): Increased reticulin fibres highlight the nodular architecture (Reticulin stain, x100). (F): Proliferated bile ducts (BD) intermingle with "intermediate-appearing" structures (IS) made of small groups of hepatocytes centred by lumina of variable size and shape (H&E, x200).*
